# The Alliance of Genome Resources: Building a Modern Data Ecosystem for Model Organism Databases

**DOI:** 10.1534/genetics.119.302523

**Published:** 2019-10-17

**Authors:** The Alliance of Genome Resources Consortium

**Keywords:** model organism databases, bioinformatics, data stewardship, database sustainability

## Abstract

Model organisms are essential experimental platforms for discovering gene functions, defining protein and genetic networks, uncovering functional consequences of human genome variation, and for modeling human disease. For decades, researchers who use model organisms have relied on Model Organism Databases (MODs) and the Gene Ontology Consortium (GOC) for expertly curated annotations, and for access to integrated genomic and biological information obtained from the scientific literature and public data archives. Through the development and enforcement of data and semantic standards, these genome resources provide rapid access to the collected knowledge of model organisms in human readable and computation-ready formats that would otherwise require countless hours for individual researchers to assemble on their own. Since their inception, the MODs for the predominant biomedical model organisms [*Mus sp*. (laboratory mouse), *Saccharomyces cerevisiae*, *Drosophila melanogaster*, *Caenorhabditis elegans*, *Danio rerio*, and *Rattus norvegicus*] along with the GOC have operated as a network of independent, highly collaborative genome resources. In 2016, these six MODs and the GOC joined forces as the Alliance of Genome Resources (the Alliance). By implementing shared programmatic access methods and data-specific web pages with a unified “look and feel,” the Alliance is tackling barriers that have limited the ability of researchers to easily compare common data types and annotations across model organisms. To adapt to the rapidly changing landscape for evaluating and funding core data resources, the Alliance is building a modern, extensible, and operationally efficient “knowledge commons” for model organisms using shared, modular infrastructure.

## A Brief History of Model Organism Databases and the Gene Ontology Consortium

BECAUSE many basic biological processes and molecular mechanisms are shared across all extant organisms, discoveries in diverse nonprimate organisms can reveal fundamental properties of the homologous biological processes in humans. Model organisms, including *Mus sp*. (laboratory mouse), *Saccharomyces cerevisiae*, *Drosophila melanogaster*, *Caenorhabditis elegans*, *Danio rerio*, and *Rattus norvegicus*, and model systems less commonly used have provided insights into the biological processes that underlie human health and disease, and have contributed to the development of diagnoses and treatments for genetic diseases (Iannaccone and Jacob 2009; Phillips and Westerfield 2014; Hamza *et al.* 2015; Kachroo *et al.* 2015; Strange 2016; Ugur *et al.* 2016; Bonini and Berger 2017; Golden 2017; Sen and Cox 2017; Wangler *et al.* 2017; Apfeld and Alper 2018; Ingham 2018; Nadeau and Auwerx 2019; Smith *et al.* 2019).

Model organism databases (MODs) have played a central role in the success of animal models in basic and biomedical research for decades by providing ready access to knowledge about genome features, their functions, and their associated phenotypes. MODs obtain and continually update this information through expert curation and integration of heterogeneous data and information from peer-reviewed scientific literature, and from direct data submissions. To assist researchers in finding appropriate models for studying biological mechanisms that contribute to complex phenotypes and disease, MODs provide access to inventories of biological reagents that are available from stock centers and strain repositories. They also maintain linkages to relevant data available in scores of other genome-centric bioinformatics resources and sequence archives, such as UniProtKB (UniProt Consortium 2019) and GenBank (Benson *et al.* 2018). The MODs work closely with their respective organism-specific research communities to define nomenclature and data format standards, and they serve as the authoritative sources of most organism-specific gene, phenotype, and disease annotations (Table 1). Acknowledgments of the MODs and the Gene Ontology Consortium (GOC) in the peer-reviewed scientific literature demonstrate that these resources are widely used to support science funded across all National Institutes of Health (NIH) Institutes and have global impact. These resources also have been leveraged heavily by bioinformatics initiatives using comparative biology approaches for functional genomics, including MARRVEL (Model organism Aggregated Resources for Rare Variant ExpLoration) (Wang *et al.* 2017), the Monarch Initiative (Mungall *et al.* 2017), GeneWeaver (Bubier *et al.* 2017), Gene2Function (Hu *et al.* 2017), and modEnrichr (Kuleshov *et al.* 2019). As noted by Oliver *et al.* (2016), “Without the systematic organization of the MODs, each of our research efforts would be drastically impeded and, in some cases, impossible, slowing the pace of discovery and reducing the efficient use of NIH funding.”

**Table 1:** The founding members of the Alliance of Genome Resources and the data for which the resource is the authoritative source: the NHGRI at the NIH is the primary funder for all of the resources except for Rat Genome Database, where the primary funding comes from the National Heart Lung Blood Institute

Genome resource	Year founded	Authoritative data/annotations
Mouse Genome Database (MGD): http://www.informatics.jax.org/; Bult *et al.* (2019)	1989	Mouse gene, allele, and strain nomenclature; gene function (GO) annotations; phenotype annotations; mouse models of human disease; unified genome feature catalog for the mouse reference genome
FlyBase: https://flybase.org/; Thurmond *et al.* (2019)	1992	*Drosophila* gene and allele nomenclature; gene function annotations (GO); protein annotation; phenotype annotations; fly models of human disease
Saccharomyces Genome Database (SGD): https://yeastgenome.org/; Cherry *et al.* (2012)	1993	*S. cerevisiae* reference genome sequence; reference proteome and chromosomal feature annotations; standardized nomenclature for gene names; gene product annotations; gene function (GO) annotations; phenotype and human disease associations; regulatory networks; gene expression patterns; Metabolic pathways; curation of all *S. cerevisiae* published literature.
Zebrafish Information Network (ZFIN): https://zfin.org/; Westerfield *et al.* (1997)	1994	Zebrafish gene, allele, and strain nomenclature; gene function (GO) annotations; gene expression annotations; phenotype annotations; zebrafish models of human disease; unified genome feature catalog for the zebrafish reference genome; reagents; catalog of Zebrafish researchers
Gene Ontology Consortium (GOC): http://geneontology.org/: The Gene Ontology (2019)	1998	GO (classification of gene functions) terms and relationships among terms for Biological Process, Molecular Function, and Cellular Component; GO annotations from multiple sources
Rat Genome Database (RGD): https://rgd.mcw.edu/; Laulederkind *et al.* (2018)	1999	Rat gene, allele, QTL, cell line, and strain nomenclature; gene function (GO) annotations; human disease, phenotype, and pathway annotations; quantitative phenotype measurement records, including expected ranges for individual rat strains.
WormBase: https://www.wormbase.org/; Lee *et al.* (2018)	2000	*C. elegans* reference genome sequence and curated gene structures; nomenclature for numerous data types, including genes and alleles; gene function (GO) annotation; expression and interaction annotation; ontologies for nematode phenotypes and development; catalog of *C. elegans* researchers.

GO, Gene Ontology.

Common needs of the different MOD user communities have led to collaborations among the MODs to develop novel and important centralized genome resources. Gene Ontology (GO), for example, was launched to annotate gene product function, biological processes, and cellular location across different organisms with common, well-defined terms (Ashburner *et al.* 2000). Start-up funding from AstraZeneca, along with stable funding provided subsequently by the National Human Genome Research Institute (NHGRI), supported the centralized development of the GO and related software tools, as well as coordinated gene function curation efforts among the first GOC members: FlyBase, the Mouse Genome Database (MGD), and the *Saccharomyces* Genome Database. The GOC has since grown to include > 30 active members (see http://geneontology.org/docs/annotation-contributors/) and is one of the most cited resources in biomedicine (Duck *et al.* 2016). It is a crucial resource for the interpretation of high-throughput experimental data, and cross-species data retrieval and aggregation (Blake and Bult 2006). The GO has also spurred the development of a number of other ontologies for related biological domains, including Cell Ontology (Diehl *et al.* 2016) and Uberon, the multi-species anatomy ontology (Mungall *et al.* 2012).

The fundamental data management principles upon which MODs and the GOC were built were designed to promote “rigor and reproducibility” in biomedical research, through the generation and maintenance of stable references to biological entities and annotations. In recent times, these concepts are better known as FAIR principles (Findability, Accessibility, Interoperability, and Reusability) (Wilkinson *et al.* 2016). These principles remain a constant at the core of operations for MODs and the GOC, even as the resources continually adapt to accommodate new data types, curation methods, and data management technologies.

## The Changing Landscape for Sustaining Core Data Resources

Although several of the current major MODs existed prior to the genome era (Table 1), a large investment was made in genome knowledgebases by the NIH and, in particular, the NHGRI, starting around the time of the Human Genome Project in the early 1990s. These investments were made in recognition of the importance of model organisms for understanding the biology of the human genome and for advancing the application of genomics to medical practice. An NIH-sponsored workshop focusing specifically on the importance of nonmammalian model organisms was held in February 1999 (see https://web.archive.org/web/20000818110738/https://www.nih.gov/science/models/nmm/) following a similar workshop organized by the National Cancer Institute in 1997 (see https://web.archive.org/web/20000818162500/http://www.nih.gov/science/models/nmm/nci_nmm_report.html). The executive summary from the 1999 meeting emphasized the critical need for genome sequencing, molecular and organismal reagents, and public databases for nonmammalian model organisms to support the interpretation of the human genome.

Early in 2016, the NHGRI, the primary funder of most MODs and the GOC, announced their intent to scale back funding for these community genome resources by 30% by Fiscal Year 2021. The leaders of the MODs and the GOC were urged by the NHGRI to restructure the organization, management, and operations of their resources to achieve substantial cost savings (Hayden 2016; Kaiser 2016). The main justifications for the mandated changes were threefold. First, there was a concern that the lack of uniformity in user interfaces across the different resources had resulted in unintentional “siloing” of information because users had to navigate different search and display options for common data types at the different MOD websites. For computational biologists, the lack of unified programmatic data access methods meant that unique code had to be written for each database to retrieve similar types of data and annotations. Second, there was a perception that there were unnecessary redundancies in operations and infrastructure, due to the independent and distributed nature of the genome resources. Centralization of infrastructure was seen as a means to reduce the overall operational and management costs of the resources. Finally, while recognizing the critical importance of these resources, the NHGRI argued that the ongoing financial commitment to these resources was restricting the investments that they could make in new areas of genome research.

In May of 2016, the principal investigators from six MODs and the GOC presented a concept for a unified MOD/GOC initiative to NIH program officials and their external scientific advisors. Following this meeting, the MOD/GOC coalition submitted a formal proposal to fund the initial steps needed to implement the proposed framework. This proposal was awarded as an administrative supplement to the WormBase grant in September of 2016, formally launching the Alliance.

The response of the research community to the NHGRI’s announcement of reduced funding for the MODs/GOC was one of concern and alarm. Under the auspices of the Genetics Society of America, the Society for Developmental Biology, and the American Society of Cell Biology, a Statement of Support for the MODs was published that urged the NHGRI/NIH to reconsider the funding cutbacks. The Statement highlighted the importance of the MODs in supporting basic research and discovery, and advocated for continued “adequate and sustained funding” for the resources. The statement was signed by over 11,000 scientists (see Poston 2016; http://genestogenomes.org/action-alert-support-model-organism-database-funding/), including 12 Nobel Laureates and 57 members of the National Academy of Sciences. It was presented to the NIH Director, Francis Collins, at The Allied Genetics Conference (TAGC) in the summer of 2016 (Organizers of The Allied Genetics Conference 2016).

As a follow-up to the TAGC meeting, NIH program officials and external advisors, community stakeholders, and representatives of the MODs and the GOC assembled for a meeting on genome resource sustainability in Bethesda in March 2017. At this meeting, the plans and progress of the Alliance were reported and discussed. Although no specific plans were presented at this meeting for a new evaluation and funding model for community resources, NHGRI Director Eric Green reported on early stage national and international discussions focused on developing strategies for sustainable funding of core data resources. Patricia Brennan, the newly appointed National Library of Medicine (NLM) Director, acknowledged the importance of MODs, and affirmed the NLM’s commitment to data standards and interoperability.

The NIH released a strategic plan for data science in June 2018 (see https://www.nih.gov/news-events/news-releases/nih-releases-strategic-plan-data-science). The plan outlines the need and vision for “modernizing the NIH-funded biomedical data science ecosystem,” addresses the challenges of defining meaningful criteria with which to evaluate core community resources, and acknowledges the need for evaluation criteria specifically for bioinformatics resources. Although the plan touches on many of the important challenges for data science in biomedical research, a corresponding tactical plan for sustainable funding of core resources has yet to emerge.

## The Alliance of Genome Resources

**Figure 1:**
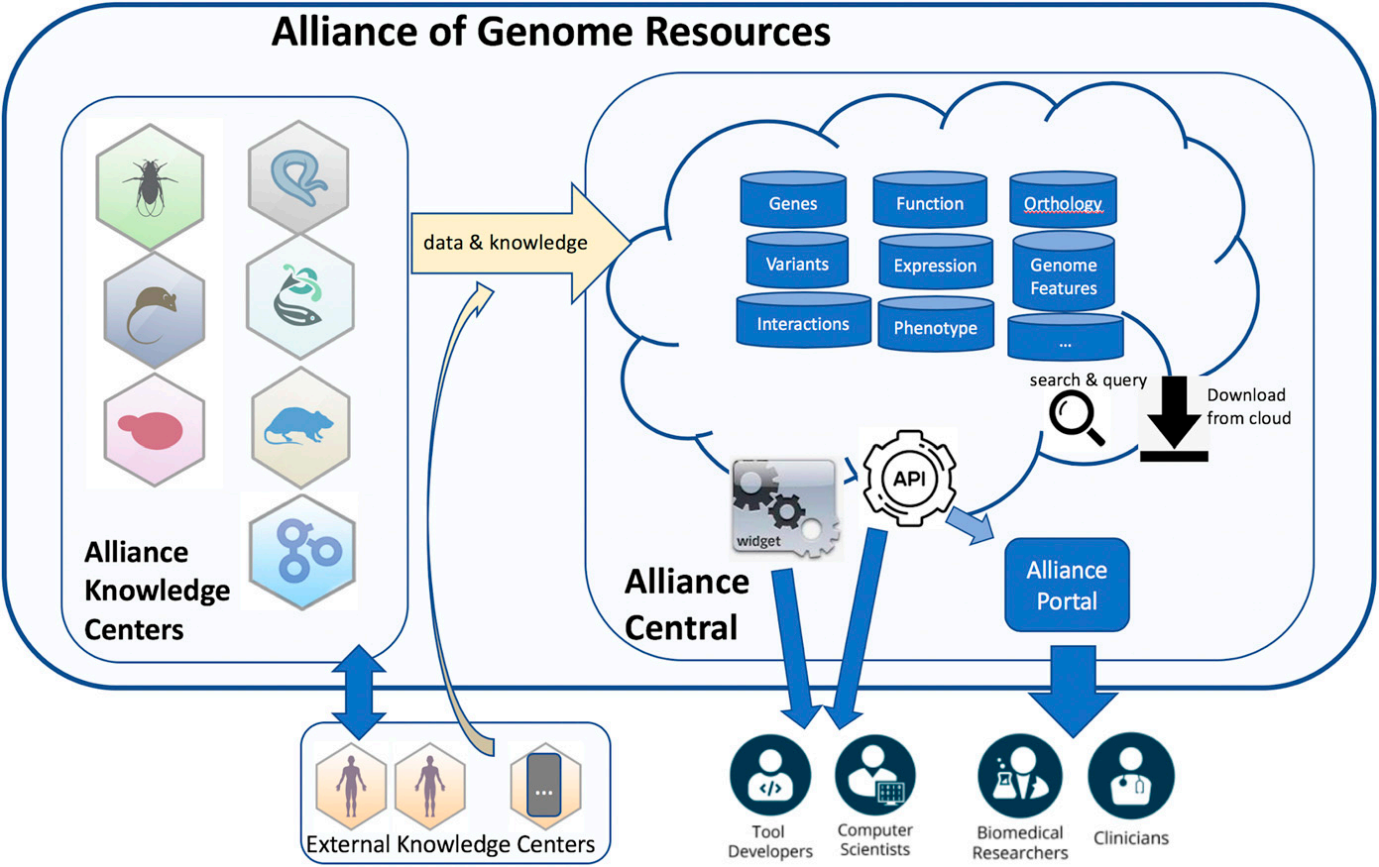
The Alliance of Genome Resources is organized into Knowledge Centers (expert curation, development of ontologies and standards, and data integration) and Alliance Central (data management and delivery, software tools, and widgets). Alliance Central provides centralized infrastructure support for Knowledge Centers. Knowledge Centers are federated to support maximally effective organism-specific data acquisition and curation. Shared standards for knowledge representation and data formats allow for unification of Alliance Knowledge Centers with external knowledge bases that are relevant to the Alliance mission but are not formal Alliance members. API, application programming interface.

The Alliance of Genome Resources is more than a formal consortium among the MODs and the GOC. It represents a significant departure from a mostly decentralized approach to knowledgebase development and maintenance to a highly centralized and coordinated effort. Organizationally, the Alliance has two interdependent functional units: *Alliance Central* and *Alliance Knowledge Centers* (Figure 1). Alliance Central is responsible for developing and maintaining the software platform and shared modular infrastructure, and for the coordination of data harmonization activities across the Knowledge Centers. The coordination of infrastructure development reduces redundancy in systems administration, software development, and ensures a unified “look and feel” for access and display of data types in common across diverse model organisms. Alliance Knowledge Centers are responsible for expert curation of data and for submission of data to Alliance Central using common standardized data formats. Knowledge Centers also are responsible for organism-specific user support activities and for providing access to data types not yet supported by Alliance Central.

The Alliance of Genome Resources serves the same diverse research communities supported by the existing collective of model organism genome resources including: (i) *human geneticists and clinical researchers* who want access to all model organism data, which are the main sources of experimental annotation of human genes through orthology; (ii) *basic scientists* who use specific model organisms to investigate fundamental biology; (iii) *computational biologists and data scientists* who need access to standardized, well-structured data, both big and small; and (iv) *educators and students*. As a consortium, the Alliance is a powerful advocate for model organism research and will serve these diverse user communities even better than before. Model organism researchers will benefit from streamlined development and coordinated delivery of access to new data types and user interfaces. Model organisms with smaller user communities will be able to leverage Alliance infrastructure to enhance their impact in advancing genome biology and translational research. Computational biologists and data scientists will benefit from the centralized data access and common Application Programming Interfaces.

Since its official launch in 2016, the Alliance has made substantial progress toward unified access to common data types across different organisms and the development of a scalable data ecosystem for model organism knowledgebases (Table 2). Examples of the accomplishments of the Alliance to date include: (i) a single integrated Alliance orthology gene set for comparative genomics of humans and model organisms, based on the work of the Quest for Orthologs Consortium (Glover *et al.* 2019); (ii) adoption of the Disease Ontology as the common annotation standard for annotating human disease association; (iii) a ribbon visualization widget to display summary annotations for gene function, phenotype, and expression developed initially by the MGD (Bult *et al.* 2016) that has been implemented by Alliance developers as a reusable web component for displaying annotations across multiple organisms, and (iv) a computational method developed by WormBase for automatically generating brief, readable summaries of gene function from ontology annotations, which is now used across the Alliance members to generate gene summaries for model organisms and human. A recent publication on the functionality currently supported by the Alliance website illustrates how researchers can search the resource by gene symbols, gene function terms, and disease terms, and then review annotations from all six model organisms and human using interfaces that share a common look and feel (Alliance of Genome Resources Consortium 2019).

**Table 2:** Examples of the accomplishments of the Alliance of Genome Resources to date in the areas of organization, process, data, and interfaces and how these accomplishments benefit the research community

Accomplishment	Community benefit
*Organization:* Common project management and governance structure	Ability to leverage unique capabilities and expertise to enhance genome resources
*Organization:* Centralized user Help Desk	Single point of access for inquiries about data for any organism in the Alliance
*Organization:* Coordinated software development	Rapid propagation of access to new data types and interfaces across model organisms
*Process:* Data harmonization	Essential for developing user interfaces with a unified “look and feel” for common data types
*Process:* Automated processes for concise, human-readable summaries of gene function	A short, human readable summary of gene function standardized across all model organisms in the Alliance
*Data:* Common set of orthologs	Supports comparisons of gene function, phenotype, and disease annotations among model organisms and with human data
*Data:* Common protein–protein interaction data	Leverage existing community resources to provide a common set of PPI data for all model organisms in the Alliance (Orchard *et al.* 2012; Oughtred *et al.* 2019)
*Interface:* Sequence display widget	Common graphical representation of transcripts for a gene
*Interface:* JBrowse genome browser	Adoption of externally developed software as the standard genome browser for all model organisms Skinner *et al.* (2009)
*Interface:* “Ribbon” widget for visualizing gene function and expression annotation summaries	Unified visualization paradigm for annotation summary information across all model organisms in the Alliance
*Interface:* Common web pages for genes and diseases	Consistent organization of common data types across all model organisms in the Alliance
*Interface:* Common application programming interface for common data types	Single point of programmatic access for common data types across all model organisms in the Alliance

## Future Directions for the Alliance and Core Data Resource Sustainability

The transformational potential of the Alliance of Genome Resources is already being realized in operational efficiencies and enhanced user experiences, driven by an enhanced capacity for rapid delivery of new data types and user interfaces designed to facilitate comparative biology. The approach to infrastructure development within the Alliance reflects the central principles articulated in the NIH’s data science strategic plan as well as the requirements for core community resources outlined by the European life-sciences Infrastructure for biological information (ELIXIR) program initiative (Durinx *et al.* 2016). The Alliance builds on previous successful cross-MOD projects and related initiatives, including the Generic Model Organism Database project (Stein *et al.* 2002; O’Connor *et al.* 2008) and InterMine (Lyne *et al.* 2015). Tools and interfaces developed by the Alliance are architected for reuse by others. The Alliance-developed “Sequence Feature Viewer” widget, for example, has been adopted by the Monarch Initiative (Mungall *et al.* 2017) for use at their website. Further, the Alliance will seek to adopt, rather than develop, tools and interfaces. For example, the Alliance is using JBrowse (Skinner *et al.* 2009) as a common genome browser application and are working with the JBrowse development team to add new functionality.

Eventually, the Alliance resource will reflect the union of data and functionality currently supported by individual MODs and the GOC, but this will take several years to achieve because it is critical that this goal be accomplished without sacrificing existing quality of service and timeliness of data updates to organism-specific user communities. For the near term, the Alliance web portal (www.alliancegenome.org) and the original, pre-Alliance MOD and GOC websites and infrastructure will coexist. Gradually, interfaces and resources developed by the Alliance are being deployed by the individual MODs. As new shared components are developed within Alliance Central, each MOD will retire its existing infrastructure and adopt the shared components. By 2024, we envision that the concept of “develop once, use by all” will be the standard operating procedure for data types and software tools shared among all Alliance Knowledge Centers.

The vision and roadmap for the Alliance are clear, and the initiative will be funded by the NHGRI for at least the next 5 years (2019–2024). However, there is significant uncertainty regarding long-term funding for the Alliance and all core community data resources. Ideas for funding models to reduce reliance on federal grants include public funding, third party payers, and commercialization (Anderson and Global Life Science Data Resources Working Group 2017; Gabella *et al.* 2017). The international nature of community resources and their user communities will likely require a mixed model to address the questions of what constitutes a core data resource, and how to sustain it.

Just as there are well-accepted principles for data management (*e.g.*, FAIR) to support data reuse, decisions regarding funding for core community data resources should be guided by principles of data stewardship that extend beyond initial funding for data generation and short-term support for project-specific data coordination centers. For data and information that are of broad utility to the research community, data stewardship practices by the agencies that fund data generation should reflect a commitment to Sustained data Access For Everyone (SAFE) and be measured by adherence to—and long-term financial support of—essential data stewardship practices (Peng *et al.* 2015), including:

- sustainable and open data access (both programmatic and web-based),
- preservation of data and annotation quality,
- maintenance of data integrity and usability,
- traceability of provenance for data and annotations,
- clear guidelines for permissible data use, and
- support for community outreach and training.

The MODs, GOC, and the Alliance are data stewards for the global research community. Our efforts ensure that best practices for data management and data stewardship principles are enforced. In turn, this work preserves, and enhances, the impact of the significant financial investment made by government agencies and foundations in biological and biomedical research initiatives.

## The Alliance of Genome Resources Consortium

Carol J. Bult

The Jackson Laboratory for Mammalian Genetics,

Bar Harbor, Maine 04609

(Orcid ID: 0000-0001-9433-210X)

Judith A. Blake

The Jackson Laboratory for Mammalian Genetics,

Bar Harbor, Maine 04609

(Orcid ID: 0000-0001-8522-334X)

Brian R. Calvi

Department of Biology, Indiana University,

Bloomington, Indiana 47405

(Orcid ID: 0000-0001-5304-0047)

J. Michael Cherry

Department of Genetics, Stanford University,

Palo Alto, California 94305

(Orcid ID: 0000-0001-9163-5180)

Valentina DiFrancesco

National Human Genome Research Institute,

Bethesda, Maryland 20892

Robert Fullem

National Human Genome Research Institute,

Bethesda, Maryland 20892

(Orcid ID: 0000-0003-0141-7767)

Kevin L. Howe

European Bioinformatics Institute (EMBL-EBI), European Molecular Biology Laboratory,

Hinxton, Cambridgeshire CB10 1SD, UK

(Orcid ID: 0000-0002-1751-9226)

Thom Kaufman

Indiana University

Bloomington, Indiana 47405

(Orcid ID: 0000-0003-1406-7671)

Chris Mungall

Lawrence Berkeley National Laboratory,

Berkeley, California 94720

(Orcid ID: 0000-0002-6601-216)

Norbert Perrimon

Department of Genetics, Harvard University,

Boston, Massachusetts 02138

(Orcid ID: 0000-0001-7542-472X)

Mary Shimoyama

Medical College of Wisconsin, Madison, Wisconsin 53226

(Orcid ID: 0000-0003-1176-0796)

Paul W. Sternberg

Division of Biology and Biological Engineering,

California Institute of Technology,

Pasadena, California 91125

(Orcid ID: 0000-0002-7699-0173)

Paul Thomas

Keck School of Medicine, University of Southern California,

Los Angeles, California 90089

(Orcid ID: 0000-0002-9074-3507)

Monte Westerfield

Department of Biology, University of Oregon, Eugene,

Oregon 97403
